# Curcumin analog WZ35 induced cell death via ROS-dependent ER stress and G2/M cell cycle arrest in human prostate cancer cells

**DOI:** 10.1186/s12885-015-1851-3

**Published:** 2015-11-06

**Authors:** Xiuhua Zhang, Minxiao Chen, Peng Zou, Karvannan Kanchana, Qiaoyou Weng, Wenbo Chen, Peng Zhong, Jiansong Ji, Huiping Zhou, Langchong He, Guang Liang

**Affiliations:** 1 School of Pharmacy, Health Science Center, Xi’an Jiaotong University, Xi’an, 710061 Shanxi China; 2 Chemical Biology Research Center, School of Pharmaceutical Sciences, Wenzhou Medical Universtiy, Wenzhou, 325035 Zhejiang China; 3 Department of Pharmacy, the First Affiliated Hospital of Wenzhou Medical University, Wenzhou, 325035 Zhejiang China; 4 Department of Interventional Radiology, The Fifth Affiliated Hospital of Wenzhou Medical University, Lishui, 323000 Zhejiang China

**Keywords:** Cell cycle arrest, CHOP, Curcumin analog, ER stress, Prostate cancer, PC-3, ROS

## Abstract

**Background:**

Prostate cancer is the most commonly diagnosed malignancy among men. The Discovery of new agents for the treatment of prostate cancer is urgently needed. Compound WZ35, a novel analog of the natural product curcumin, exhibited good anti-prostate cancer activity, with an IC_50_ of 2.2 μM in PC-3 cells. However, the underlying mechanism of WZ35 against prostate cancer cells is still unclear.

**Methods:**

Human prostate cancer PC-3 cells and DU145 cells were treated with WZ35 for further proliferation, apoptosis, cell cycle, and mechanism analyses. NAC and CHOP siRNA were used to validate the role of ROS and ER stress, respectively, in the anti-cancer actions of WZ35.

**Results:**

Our results show that WZ35 exhibited much higher cell growth inhibition than curcumin by inducing ER stress-dependent cell apoptosis in human prostate cells. The reduction of CHOP expression by siRNA partially abrogated WZ35-induced cell apoptosis. WZ35 also dose-dependently induced cell cycle arrest in the G2/M phase. Furthermore, we found that WZ35 treatment for 30 min significantly induced reactive oxygen species (ROS) production in PC-3 cells. Co-treatment with the ROS scavenger NAC completely abrogated the induction of WZ35 on cell apoptosis, ER stress activation, and cell cycle arrest, indicating an upstream role of ROS generation in mediating the anti-cancer effect of WZ35.

**Conclusions:**

Taken together, this work presents the novel anticancer candidate WZ35 for the treatment of prostate cancer, and importantly, reveals that increased ROS generation might be an effective strategy in human prostate cancer treatment.

**Electronic supplementary material:**

The online version of this article (doi:10.1186/s12885-015-1851-3) contains supplementary material, which is available to authorized users.

## Background

Prostate cancer is the most commonly diagnosed malignancy among men in industrialized countries, accounting for the second leading cause of cancer-related death. Conventional therapies produce a high rate of cure for patients with localized prostate cancer by surgical therapy, but there is no cure once the disease has spread beyond the prostate. Traditionally, the treatment of prostate cancer has been based on the deprivation of androgens to the developing tumor. Though initially successful, this form of therapy fails in advanced stages of the disease, as the cells develop the ability to sustain growth and proliferation even in the absence of androgens, thus acquiring androgen resistance [1]. In addition, these tumors tend to be highly resistant to conventional cytotoxic agents such as cisplatin. Presently available treatments for advanced hormone-resistant prostate cancer are marginally effective; thus, new agents are needed to selectively kill cancer cells.

**Fig. 1:**
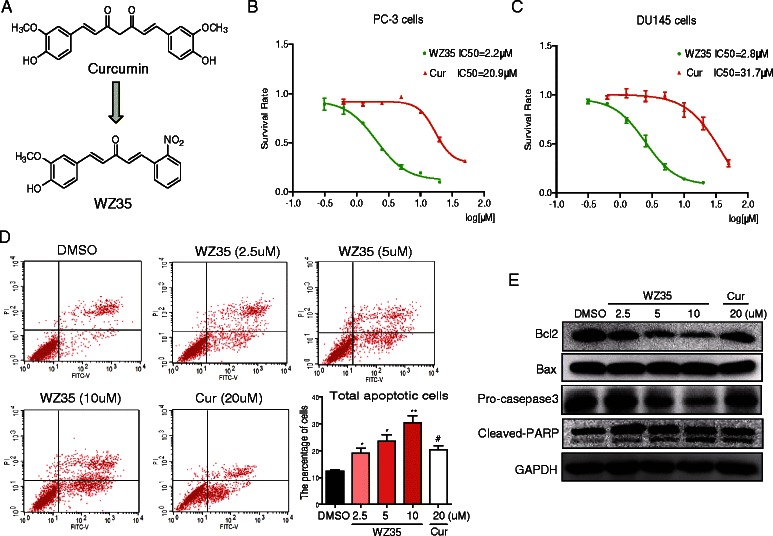
Effects of curcumin analog WZ35 on cell viability and apoptosis in human prostate cancer cells. **a** The chemical structure of curcumin and curcumin analog WZ35. **b**–**c** The effects of WZ35 or curcumin on cell viability in human prostate cancer cells. PC-3 cells or DU145 cells were treated with WZ35 or curcumin at different concentration ranges as indicated for 48 h, then cell viability was determined by MTT assay, the IC_50_ was indicated. **d** Representative images for cell apoptosis stained with Annexin V-FITC/PI. Cells were treated with WZ35 at different concentrations as indicated or curcumin (20 μM) for 24 h, then cells were stained with Annexin V-FITC/PI and analyzed by flow cytometry as described in methods. **e** Western blot analysis for expression of apoptosis-associated proteins in cells treated with or without WZ35 or curcumin. Cells were treated with WZ35 at different concentrations as indicated or curcumin (20 μM) for 24 h, the cell lysates were processed for western blot analysis for protein expression of Bcl-2, Bax, pre-caspase 3, cleaved-PARP, and GAPDH used as a loading control. The statistic data were presented as mean ± S.E from three independent experiments. *, *p* < 0.05, **, *p* <0.01; all versus DMSO group

Curcumin, a polyphenolic compound that is extracted from the rhizome of the plant Curcuma longa, has become a focus of interest regarding its antitumor effects in multiple cancer cell types including prostate cancer cells [2]. Moreover, curcumin is under clinical trials mainly for cancer related diseases [3, 4]. Interestingly, phase1 clinical trials already demonstrated the safety of curcumin even at high doses (12 g/day). However, the clinical advancement of this promising natural compound is hampered by its poor water solubility and short biological half-life, resulting in low bioavailability in both plasma and tissues [5]. Multiple approaches are being sought to overcome these limitations. In the past several years, our lab has focused on the chemical modification of curcumin to find novel molecules for drug development [6, 7]. Previously, a series of mono-carbonyl analogs of curcumin were synthesized and evaluated against prostate cancer cells. Among them, compound WZ35 (Fig. 1a) exhibited good anti-prostate cancer activity at the cellular level, with an IC_50_ of 2.2 μM, compared to that of curcumin at 20.9μM in PC-3 cells (Fig. 1b), an androgen-resistant and high metastatic potential human prostate cancer cell lines.

Oxidative stress plays an important role in controlling cancer cell behavior. Cancer cells may potentially benefit from oxidative stress induction and the production of reactive oxygen species (ROS), which are known to increase the rate of mutations [8, 9]. However, the oxidative stress response is a balance between pro-survival and pro-apoptotic signaling pathways [10]. An uncontrolled high-level ROS also triggers a series of pro-apoptotic signaling pathways, including endoplasmic reticulum (ER) stress and mitochondrial dysfunction, and ultimately leads to cellular apoptosis [10]. Because cancer cells have a higher level of oxidative stress than non-malignant cells, they are vulnerable to the acute induction of oxidative stress that is caused by agents inducing ROS [9, 11]. Mounting evidence suggests that increasing oxidative stress might be an effective strategy to eliminate cancer cells. Increased ROS generation and oxidative stress have been reported in prostate cancer cells [11]. Thus, agents that can induce ROS generation may be effective in killing prostate cancer cells.

The aim of this study was to determine the effect and mechanism of WZ35 against prostate cancer cells. Our data demonstrate that WZ35 showed strong antitumor potential against PC-3 cells by activating ROS production and subsequently inducing ER stress-dependent apoptosis and cell cycle arrest.

## Methods

### Reagents

WZ35 (>98 % purity) was prepared in our lab using a previously described method. Curcumin, N-acetylcysteine (NAC), glutamine (L-GSH), dimethylsulfoxide (DMSO) and methyl thiazolyl tetrazolium (MTT) were obtained from Sigma-Aldrich (St. Louis, MO). The primary antibodies, including anti-Bcl2 (sc-492), anti-Bax(sc-493), anti-caspase 3 (sc-32577), anti-Cdc2 (sc-54), anti-Cyclin B1 (sc-245), anti-MDM2 (sc-965), anti-GAPDH (sc-32233), anti-p-PERK (sc-32577), horseradish peroxidase (HRP)-conjugated (sc-2313) and phycoerythrin (PE)-conjugated (sc-3755) secondary antibodies were purchased from Santa Cruz Biotechnology (Santa Cruz, CA). The primary antibodies, including anti-cleaved PARP (5625S), anti-p-eIF2α (3398S), anti-ATF4 (11815S), and anti-CHOP (2895S), were purchased from Cell Signaling Technology (Danvers, MA). CHOP siRNA was purchased from GenePharma (Shanghai, China). FITC Annexin V apoptosis Detection Kit I and propidium iodide (PI) were obtained from BD Pharmingen (Franklin Lakes, NJ). Bradford protein assay kit, polyvinyldene fluoride membrane, ECL kit were obtained from Bio-Rad (Hercules, CA). Lipofectamine 2000, TRIZOL reagent, M-MLV Reverse Transcriptase Kit, PCR Supermix kit and primers for genes, including CHOP and β-actin, were purchased from Invitrogen Life Technology (Carlsbad, CA). DCFH-DA was obtained from Beyotime Biotech (Nantong, China).

### Cell culture

Human prostate cancer PC-3 cells and DU145 cells were obtained from the Shanghai Institute of Life Sciences Cell Resource Center (Shanghai, China) and cultured in DMEM/F12 medium (Gibco, Eggenstein, Germany) that was supplemented with 10 % heat-inactivated FBS (Hyclone, Logan, UT), 100 U/mL penicillin and 100 μg/mL streptomycin (Mediatech Inc., Manassas, VA) in a humidified atmosphere of 5 % CO_2_ at 37 °C.

### Methyl Thiazolyl Tetrazolium (MTT) assay

All of the experiments were carried out 24 h after the cells were seeded. The tested compounds were dissolved in DMSO and diluted with DMEM/F12 medium at different concentrations. The tumor cells were incubated with test compounds for 48 h before the MTT assay. A fresh solution of MTT (5 mg/mL) that was prepared in PBS was added to each single well of the 96-well plate. The plate was then incubated in a CO_2_ incubator for 4 h. Formazan cyrstals that formed in living cells was dissolved in 150 μL of dimethyl sulfoxide, and the absorbance of the solution was measured at 490 nm using a microplate reader *(Reader 400 SFC, LabInstruments, Hamburg,Germany)*. The IC_50_ values were calculated using the GraphPad Prism 5 software.

### Measurement of cell apoptosis

Apoptosis was analyzed by Annexin V-FITC/PI staining. Briefly, after treatment, the cells were harvested and washed with PBS followed by the addition of 1× binding buffer (500 μl) and Annexin V-FITC (2 μl), incubated at RT in the dark for 20 min and centrifuged. The cell pellet was re-suspended in 1× binding buffer, added with 3 μl of PI (30 μg/ml) and acquired immediately on an FACS Caliber flow cytometer (BD Biosciences, CA). An analysis was performed for Annexin V-FITC binding using the FITC signal detector (FL-1) and PI staining by the phycoerythrin emission signal detector (FL-2) using the CellQuest™ software (BD Biosciences, CA) or the FlowJo 7.6 software (TreeStar, San Carlos, CA).

### Western blot assay

After treatment, the cells were collected and extracted for total proteins. The protein concentrations in all of the samples were determined using the Bradford protein assay kit. Protein samples (30–100 μg) were subjected to (10–15 %) sodium dodecyl sulfate-polyacrylamide gel electrophoresis, and transferred onto polyvinyldene fluoride membrane. After being blocked in blocking buffer (5 % milk in tris-buffered saline containing 0.05 % Tween 20) for 1.5 h at room temperature, membranes were incubated with different primary antibodies overnight at 4 °C. Then, the membranes were washed in TBST and reacted with secondary horseradish peroxidase-conjugated antibody for 1 h at room temperature, and the immunoreactive bands were visualized using an ECL kit. The density of the immunoreactive bands was analyzed using Image J computer software (National Institute of Health, MD).

### Determination of Reactive Oxygen Species (ROS) production

Intracellular ROS generation was monitored by an FACS Caliber flow cytometer (BD Biosciences, CA) using the peroxide-sensitive fluorescent probe 2′,7′-dichlorofluorescin diacetate (DCFH-DA) as previously described [12]. In brief, after treatment, the cells were incubated with 10 μM DCFH-DA at 37 °C for 30 min, resuspended in ice-cold phosphate buffered saline (PBS) and placed on ice in a dark environment. The intracellular peroxide levels were measured by an FACS Caliber flow cytometer that emitted a fluorescence signal at 525 nm. Each group was acquired for 10,000 individual cells using the CellQuest™ software (BD Biosciences, CA) and analyzed by the FlowJo 7.6 software (TreeStar, San Carlos, CA).

### RT-qPCR assay

The total mRNA was isolated from cells using TRIZOL Reagent according to the manufacturer’s instructions. Reverse transcription and quantitative PCR were performed using the M-MLV Reverse Transcriptase Kit and PCR Supermix kit according to the manufacturer’s instructions. Real-time qPCR was carried out using the Eppendorf Real plex 4 instrument (*Eppendorf, Hamburg, Germany*). The relative amount of each gene was normalized to the amount of β-actin. The primer sequences used were shown as followed. Human CHOP: forward, CAGAACCAGCAG AGGTCACA; reverse, GCTGTGCCACTTTCCTTTC. Human β-action: forward, TCCTTCCTGGGCATGGAGTC; reverse, GTAACGCAACTAAGTCATAGTC.

### Transient transfection of small interfering RNA (siRNA)

The cells were transfected with siRNA (50pmol/ml) targeting CHOP or non-targeted siRNA as a control using the lipofectamine 2000 reagent as described by the manufacturer’s instructions. Subsequently, the transfected cells were washed and changed with complete media and used in further studies. The siRNA sequences used were as followed: Human control siRNA (CtrlsiRNA): sense, 5′-AGUACUGCUUACGAUACGGTT-3′; antisense, 5′-CCGUAUCGUAAGCAGUACUTT-3′. CHOP siRNA: sense, 5′-CCAGGAAACGGACAACAGAGTT-3′; antisense, 5′-CUCUGUUUCCGUUUCCUGGTT-3′. These siRNA sequences were adopted according to previous published work [13].

### Cell cycle analysis

The cell cycle status and nuclear DNA contents were determined using propidium iodide (PI) staining and flow cytometry. Briefly, the cells were collected, fixed with 75 % ice-cold ethanol and stored at 4 °C for 24 h. After they were washed with PBS, the cells were stained with PI [50 μg/ml PI and 10 μg/ml ribonuclease (RNase) in PBS] at 4 °C for 20 min in the dark. The cells were washed and subjected to an FACS Caliber flow cytometric analysis of the DNA content. The cell fractions in the G2/M phase were used for statistical analysis using the FlowJo 7.6 software *(TreeStar, San Carlos, CA)*.

### Immunofluorescence assay for CHOP

Cells were fixed with 4 % paraformaldehyde and permeabilized with 100 % methanol at -20 °C for 5 min. After fixation and permeabilization, the cells were washed twice with PBS containing 1 % BSA and then incubated with the primary antibody for CHOP overnight at 4 °C, followed by incubation with the PE-conjugated secondary antibody. Then, the cells were counterstained with DAPI and viewed under a Nikon fluorescence microscope (400× amplification; *Nikon, Japan*).

### Statistical analysis

All of the experiments were performed independently three times. The data are presented as means ± SE. The statistical significance of differences between groups was obtained by the *student’s t*-test or ANOVA multiple comparisons in GraphPad Pro (*GraphPad, San Diego, CA*). Differences were considered significant at *, *P* < 0.05; **, *P* <0.01; ***, *P* <0.001.

## Results

### WZ35 reduced cell viability and induced cell apoptosis in human prostate cancer cells

To determine the cytotoxic effects of WZ35 in prostate cancer cell lines, an MTT assay was performed to evaluate the viability in human prostate cancer PC-3 and DU145 cells. As shown in Fig. 1b and c, WZ35 or curcumin treatment significantly decreased the viability of PC-3 cells and DU145 cells in a dose-dependent manner. The IC_50_ value for WZ35 and curcumin was 2.2 μM versus 20.9 μM in PC-3 cells, and 2.8 versus 31.7 μM in DU145 cells, respectively, indicating a much better anti-cancer ability of WZ35 than curcumin. We then evaluated the role of apoptosis in WZ35-induced cell death using Annexin V-FITC/PI staining. A time-course assay revealed that the occurrence of cell apoptosis induced by WZ35 began at 12 h and peaked at 24 h after WZ35 treatment (Additional file 1: Figure S1A). The exposure of PC-3 cells to WZ35 at various concentrations for 24 h dose-dependently increased the number of apoptotic cells (Fig. 1d). In addition, WZ35 was dramatically more effective than curcumin in apoptosis induction. The levels of apoptosis-associated proteins were also examined by western blot analysis in PC-3 cells. As shown in Fig. 1e, WZ35 treatment decreased the protein level of Bcl-2 and pro-caspase 3 and increased the cleaved PARP in a dose-dependent manner but had no effect on Bax expression. No obvious changes were observed in these protein levels in cells that were treated with 20 μM curcumin (Fig. 1e).

### ROS overproduction mediated WZ35-induced apoptosis in PC-3 cells

**Fig. 2:**
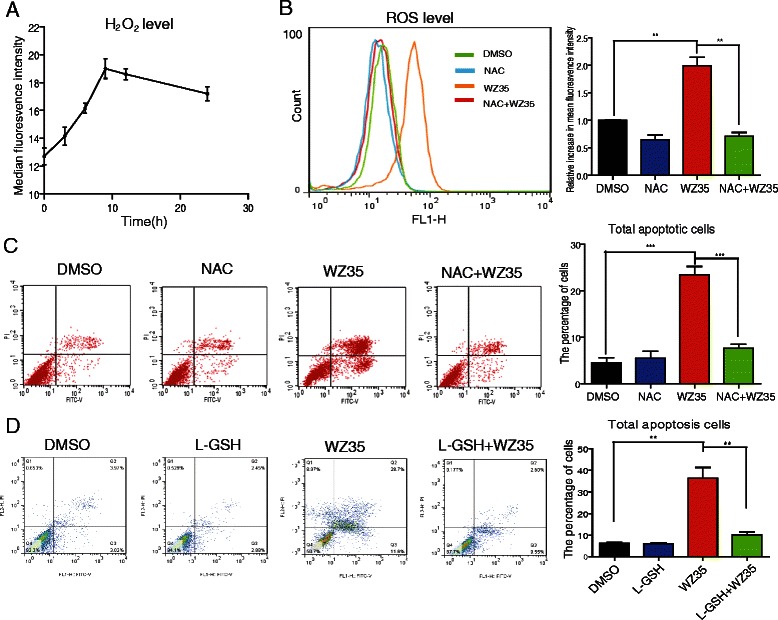
WZ35 induced cell apoptosis is via oxidative stress. **a** The time-course ROS generation induced by WZ35. Cells were treated with WZ35 (10 μM) for different time as indicated, then cells were strained with DCFH-DA and the DCF fluorescence intensity was analyzed with flow cytometry. **b** Representative image for ROS generation in cells treated with WZ35 in the presence or absence of NAC. Cells were treated with WZ35 (10 μM) in the presence or absence of NAC (10 mM) for 9 h, then cells were stained with DCFH-DA and DCF fluorescence intensity was analyzed by flow cytometry as described in methods. The relative increase in DCF fluorescence intensity was indicated. **c**–**d** Representative images for cell apoptosis stained with Annexin V-FITC/PI. Cells were treated with WZ35 (10 μM) in the presence or absence of NAC (10 mM) or L-GSH (10 mM) for 24 h, then cells were stained with Annexin V-FITC/PI, and analyzed by flow cytometry as described in methods. The statistic data were presented as mean ± S.E from three independent experiments. **, *p* <0.01; ***, *p* <0.001

We investigated whether intracellular ROS generation was implicated in the anti-cancer effects of WZ35. The ROS level was assessed by using fluorescent probe DCFH-DA that detected H_2_O_2_. Interestingly, WZ35 treatment significantly increased intracellular ROS generation in a time-dependent manner (Fig. 2a). Furthermore, co-treatment with N-acetylcysteine (NAC), an ROS scavenger, significantly inhibited WZ35-induced ROS generation (Fig. 2b). These data show that WZ35 could induce the accumulation of ROS in prostate cancer cells. We then examined whether increased ROS was required for cell apoptosis induced by WZ35. As shown in Fig. 2c, co-treatment with NAC at the concentration of 10 mM almost completely abrogated WZ35-induced cell apoptosis. In addition, reversed cell apoptosis was also observed in WZ35-treated cells in the presence of glutathione (L-GSH), another potent antioxidant that has been widely used to define the function of ROS in numerous biological and pathological processes (Fig. 2d). Collectively, these results indicated that ROS generation plays a central role in mediating WZ35-induced cell apoptosis.

### WZ35-induced cell apoptosis through ER stress-mediated CHOP expression in PC-3 cells

**Fig. 3:**
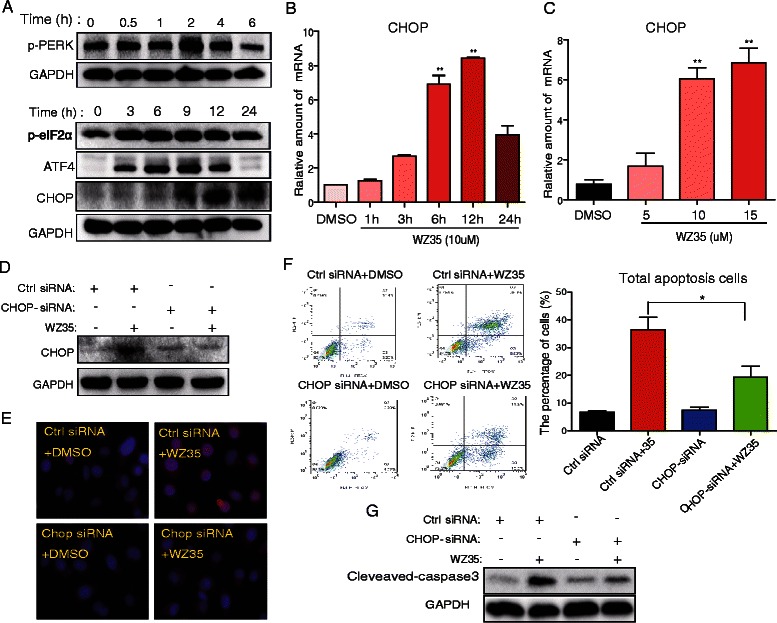
WZ35 induced cell apoptosis through ER stress-mediated CHOP expression in PC-3 cells. **a**–**b** The time-course expression of ER stress markers induced by WZ35. Cells were treated with WZ35 (10 μM) at different time interval as indicated, then cells were processed for western blot analysis or RT-qPCR for expression of ER stress markers such as p-PERK, p-eIF2α, ATF4, and CHOP as described in methods, and GAPDH or β-actin was used as a loading control. **c** The mRNA expression of CHOP in cells treated with WZ35. Cells were treated with WZ35 at different concentrations as indicated for 12 h, then the mRNA expression of CHOP was detected with RT-qPCR assay as described in methods, and β-actin was used as a loading control. **d**–**g** The effects of CHOP-siRNA on cell apoptosis induced by WZ35. Cells were transfected with Ctrl siRNA or CHOP siRNA for 24 h as described in methods. Then the transfected cells were treated with or without WZ35 (10 μM) for 24 h. Then the cells were processed for western blot analysis for CHOP expression (**d**) or processed for CHOP immunofluoresence staining (**e**) or stained with Annexin V-FITC/PI followed by flow cytometry analysis (**f**) or processed to western blot analysis for cleaved-caspase3 expression (**g**), as described in methods. The statistic data were presented as mean ± S.E from three independent experiments. *, *p* <0.05, versus Ctrl siRNA + WZ35 group

ROS generation has been reported to activate multiple pro-apoptotic cascades, including the ER stress-induced cancer cell apoptosis pathway [10, 14]. Thus, we determined the effects of treatment with WZ35 on the induction of ER stress. When PC-3 cells were treated with WZ35 for various time intervals, we noticed a transient increase in the level of phosphorylated PERK, commencing after 2 h of treatment with WZ35 and remaining elevated for up to 4 h (Fig. 3a). WZ35 treatment also induced a constant increase in the level of phosphorylated eIF2α 3 to 12 h after WZ35 treatment (Fig. 3a). ATF4 expression also increased in a similar manner with p-eIF2α (Fig. 3a).

CHOP is considered a marker of the commitment of ER stress-induced apoptosis. Western blotting analysis further showed that CHOP protein expression apparently increased 9-24 h after WZ35 treatment and peaked at 12 h (Fig. 3a). Similar time-course results were also observed in the mRNA level of CHOP induction (Fig. 3b). Figure 3c shows that compound WZ35 induced CHOP mRNA up-regulation in a dose-dependent manner. These results suggest that WZ35 can induce ER stress in prostate cancer cells. In order to further confirm that ER stress plays an important role in the induction of PC-3 cell apoptosis by WZ35, we constructed the siRNA for CHOP gene silencing. PC-3 cells were transfected with the CHOP siRNA sequence or the control sequence. Western blot analysis demonstrated that the transfection of CHOP siRNA resulted in a significant decrease in CHOP expression in WZ35-treated PC-3 cells (Fig. 3d), compared to cells that were transfected with control scrambled siRNA. This result was further confirmed by immunofluorescence staining (Fig. 3e). Furthermore, to confirm that a reduction of CHOP expression inhibits WZ35-induced PC-3 cell apoptosis, we treated CHOP siRNA-transfected PC-3 cells with WZ35. Figure 3f shows that when CHOP expression in PC-3 cells was silenced, cell apoptosis induced by WZ35 was significantly reduced compared to that of the control group (*P* < 0.05). Furthermore, the protein level of cleaved-caspase 3 induced by WZ35 was also reduced in CHOP-knockdown PC3 cells (Fig. 3g). Taken together, these results indicate that WZ35-induced cell apoptosis is, at least partly, mediated by the ER stress pathway.

### WZ35 induced cell cycle arrest in G2/M phase in PC-3 cells

**Fig. 4:**
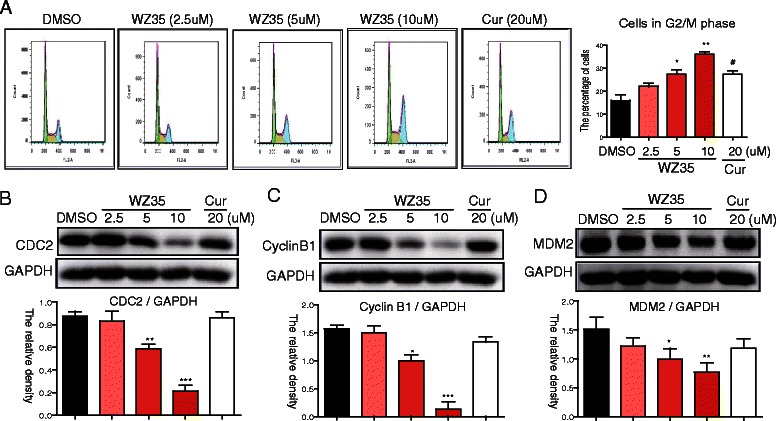
WZ35 induced cell cycle arrest in the G2/M phase in PC-3 cells. Cells were treated with WZ35 at different concentrations as indicated or curcumin (20 μM) for 24 h, then cells were processed for cell cycle analysis (**a**) or western blot analysis for expression of cell cycle-associated proteins (**b**–**d**) as described in methods. The statistic data were presented as mean ± S.E from three independent experiments. *, *p* < 0.05, **, *p* <0.01; ***, *p* <0.001; all versus DMSO group

To determine the anti-mitogenic effect of WZ35, we performed a cell-cycle analysis in PC-3 cells. As shown in Fig. 4a, WZ35 treatment induced the accumulation of cells in the G2/M phase in a dose-dependent manner. The effect of 10 μM WZ35 on cell cycle arrest was stronger than that of curcumin at 20 μM (Fig. 4a). In addition, the cell population in S phase were reduced, in association with the increased cell population in the G2/M phase, and no changes were observed in the G0 phase (Additional file 1: Figure S1B). We further tested the effects of WZ35 on cell cycle arrest-related proteins by western blot analysis. G2/M transition is regulated by the cyclin B1/CDC2 complex [15], and MDM2 is a negative regulator of p21, which is involved in the G2/M checkpoint and is required for cell cycle arrest in the G2/M2 phase [16, 17]. Figure 4b–d reveals that WZ35 treatment decreased the protein level of CDC2, Cyclin B1, and MDM2 in a dose-dependent manner, while curcumin treatment at 20 μM has no significant effects on these proteins. These results suggest an anti-mitogenic effect of WZ35 in PC-3 cells.

### Both ER stress and G2/M arrest induced by WZ35 were mediated by ROS generation in PC-3 cells

**Fig. 5:**
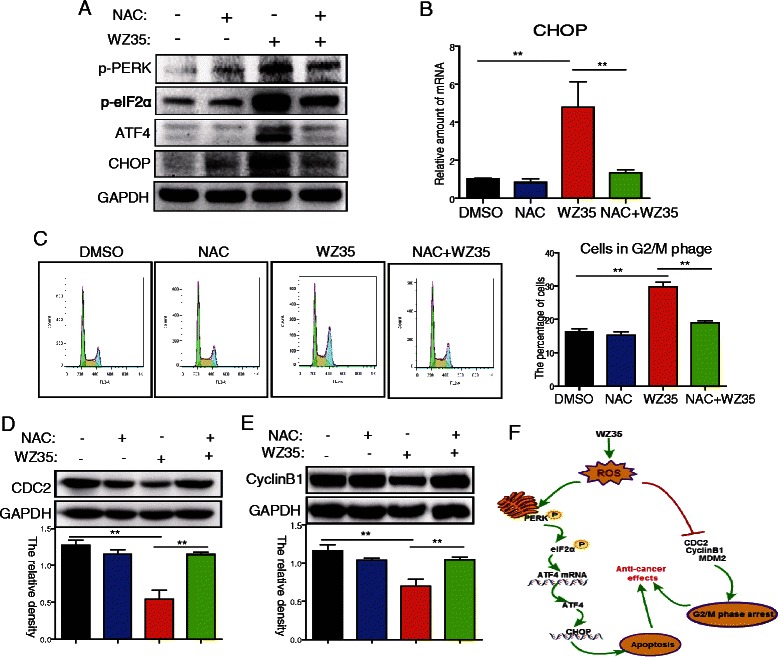
Both ER stress and G2/M arrest induced by WZ35 were mediated by ROS generation. **a**–**b** The ROS scavenger NAC reversed WZ35-induced ER stress. Cells were treated with WZ35 (10 μM) in the presence or absence of NAC (10 mM) for 24 h, then cells were processed for western blot analysis or RT-qPCR assay for the expression of ER stress markers as described in methods. **c**–**e** The ROS scavenger NAC reversed WZ35-induced G2/M arrest. Cells were treated with WZ35 (10 μM) in the presence or absence of NAC (10 mM) for 24 h, then cells were processed for cell cycle analysis (**c**) or western blot analysis for expression cell cycle-associated proteins (**d**–**e**) as described in methods. **f** The mechanism scheme of the anti-prostate cancer effects of WZ35. The statistic data were presented as mean ± S.E from three independent experiments. *, *p* < 0.05, **, *p* <0.01; all versus WZ35 group

Because ROS production induced by WZ35 occurs within a relatively much earlier time (30 min), compared to ER stress activation and cell cycle arrest, we supposed that ROS generation might be the upstream incidence in the procedure of WZ35-induced PC-3 cell death. Thus, we determined whether ROS generation is required for WZ35-induced ER stress and G2/M arrest in PC-3 cells. As shown in Fig. 5a, co-treatment with the ROS scavenger NAC markedly inhibited the WZ35-induced over-expression of ER stress markers. A similar result was also observed at the mRNA level of CHOP induced by WZ35 in the presence of NAC (Fig. 5b). These results indicate that WZ35-induced ER stress is mediated by increased oxidative stress. As expected, co-treatment with NAC also significantly inhibited the WZ35-induced accumulation of cells in the G2/M phase (Fig. 5c). In agreement, western blot analysis revealed that co-treatment with NAC significantly reversed the decreased protein level of CDC2 and CyclinB1 induced by WZ35 in PC-3 cells (Fig. 5d and e).

## Discussion

In the present study, we demonstrated that a new novel curcumin analog WZ35 showed excellent anticancer effects in prostate cancer cells via inducing ROS-dependent ER stress and G2/M cell cycle arrest. These findings indicate that WZ35 should be further explored as an effective anticancer agent for the treatment of prostate cancer. Accumulating evidence suggests that increasing oxidative stress might be an effective strategy to eliminate cancer cells [18]. Agents with the potential to induce ROS generation have anticancer effects in prostate cancer cells, such as salinomycin [19], Diallyl trisulfide [20] and WZC02-9 [21]. Herein, increased ROS generation was also observed in WZ35-treated PC-3 cells (Fig. 2). Importantly, the abrogation of ROS production by NAC co-treatment almost completely reversed the WZ35-induced cell apoptosis, suggesting the significant involvement of ROS in WZ35-induced cell death. Thus, our results further indicate that developing agents with inducing ROS potential will be a good strategy for cancer therapy.

Recently, ER stress-induced cancer cell apoptosis has become a novel signaling target for the development of cancer therapeutic drugs [22–24]. Various pathological conditions, such as hypoxia, ER-Ca2^+^ depletion, oxidative injury, hypoglycemia and viral infections, may cause an imbalance between the protein folding load and capacity; this cellular condition is known as ER stress [25]. The initial role of ER stress is tailored to re-establish ER homeostasis. However, when ER stress is too severe or cannot be solved, it changes from a pro-survival to a pro-death response, culminating in the activation of intrinsic apoptosis [26]. The most important pathway translating the cell from ER stress to death is the PERK/eIF2α pathway [14]. The phosphorylation of PERK/eIF2α subsequently induces the expression of transcription factors ATF4 and CHOP, which are important elements triggering the pro-apoptotic signaling [27]. Here, we found that WZ35 induced PERK/eIF2α activation and ATF4/CHOP expression in a time-course manner, indicating that WZ35 activated pro-apoptotic ER stress signaling (Fig. 3a–c). Then, we found that WZ35-mediated apoptosis was partially reduced in CHOP-deficient cells, confirming the mediation of ER stress in WZ35-induced cell apoptosis (Figure F). Similar to that of WZ35, targeting ER stress to induce cancer cell death has been reported by our previous works for other monocarbonyl analogs of curcumin such as B19 [28], B63 [29] and B82 [30].

Increased oxidative stress is linked to ER stress activation [25]. ROS could exacerbate protein misfolding in the ER lumen by oxidizing amino acids in folding proteins or by modifying chaperone and/or ERAD function, thereby amplifying unfolding protein response (UPR) signaling [31]. Increased ROS generation or oxidative stress may be responsible for subsequent ER stress and ER stress-dependent cell apoptosis. Antioxidants reduce ER stress and improve cell survival [32]. However, an inverse relationship between oxidative stress and ER stress has also been reported by Jypti D et al. [32]. The authors found that the accumulation of unfolded protein in the ER lumen is sufficient to produce ROS and suggested that unfolded protein in the ER lumen signalsROS production as a second messenger to activate the UPR and induce apoptosis [32]. In this study, we found that ER stress activated by WZ35 was almost completely reversed by the presence of NAC, a ROS scavenger (Fig. 5). In addition, WZ35 treatment activated ROS generation within 30 min, much earlier than the treatment time for ER stress induction. Thus, these results indicate that the increased ROS generation induced by WZ35 is required for the induction of ER stress and triggers ER stress-dependent apoptosis in PC-3 cells.

The present study also shows that WZ35 treatment inhibited G2/M progression in prostate cancer cells, in association with the decreased expression of CDC2, cyclinB1, and MDM2 (Fig. 4). As each phase of the cell cycle is driven by specific CDKs, the CDK1 (also named CDC2)-cyclin B1 complex is responsible for driving cells through mitosis [15]. MDM2 is an oncoprotein that can regulate the cell cycle and is a negative regulator of p21, which is required for cell cycle arrest in the G2/M2 phase [16, 17]. Several studies have reported that some compounds inducing ROS generation, including diallyl trisulfide [23], plumbagin [33], and diallyl disulfide [25], could induce cell cycle arrest at the G2/M phase in several cancer cells. Here, we also found the WZ35-induced G2/M arrest was reversed by co-treatment with an ROS scavenger (Fig. 5). These results suggest a strong link between oxidative stress and cell cycle arrest. Although this link has been also observed in other anti-cancer therapies, the underling mechanism has not been totally resolved. Some studies reported that p21 plays a vital role in mediating ROS-induced G2/M arrest [34]. In addition, a p21-independent mechanism has also been reported [20]. Xiao et al. revealed that increased ROS can induce the destruction and hyperphosphorylation of CDC25c, a phosphatases that can dephosphorylate CDK1 in Thr14 and Tyr15, and hence activate the CDK1/cyclinB1 kinase complex [20]. Although our study demonstrates that the ROS-induced cell cycle arrest is associated with CDC2/cyclin B1 reduction, the exact mechanism requires further research.

The excellent anticancer effects of WZ35 suggest the potential advantages of the mono-carbonyl structure of curcumin. Our group has been engaged in designing and discovering new small molecules from natural curcumin. Previously, some other monocarbonyl analogs of curcumin, such as B19 [28], B63 [29], and B82 [30], that were synthesized by our lab were demonstrated to possess strong anti-cancer effects in various cancer cells. In addition, we investigated the mechanism by which these compounds induce cancer cell death. As previously reported, one common mechanism underling their anticancer action is that all of these agents could induce lethal ER stress. This result is also observed in the anti-cancer effects of WZ35. However, our previous studies have failed to demonstrate how curcumin analogs activate ER stress. In the present study, we found that WZ35-increased ROS generation occurred upstream of ER stress. This mechanism may be also suitable for previously reported mono-carbonyl analogs of curcumin.

In addition, similar results were observed in DU145 cells. As shown in Additional file 1: Figure S2, we found that WZ35 also induced ROS accumulation, CHOP expression, cell apoptosis and G2/M arrest in DU145 cells. More importantly, all of these alterations were significantly inhibited by ROS scavenger NAC pretreatment, and the genetic silence of CHOP by siRNA approach also remarkably prevented cell apoptosis in DU145 cells. Collecting the data from DU145 and PC-3 cells, our results vividly demonstrate the pivotal role of the ROS-ER stress signaling pathway in mediating the anticancer effects of WZ35 in prostate cancer cells.

## Conclusions

In summary, a new monocarbonyl analog of curcumin, WZ35, exhibited antitumor effects on human PC-3 and DU145 cells in vitro by inducing ROS generation and subsequent ER stress and G2/M cycle arrest. The discovery of the activation of ROS-mediated apoptosis by the curcumin analog WZ35 may provide new strategy for curcumin-based anticancer drug design and development. The new compound WZ35 could be further explored as a potential anticancer agent for the treatment of prostate cancer.

## Additional file

Additional file 1:
**The anti-cancer effects of WZ35 treatment in PC-3 and DU145 cells.** **Figures S1**, The time-course of cell apoptosis and cell cycle progression in response to WZ35 treatment in PC-3 cells. and S2, The effects of WZ35 treatment in DU145 cells.(DOC 290 kb)
