# Supplementary material for: Curcumin analog WZ35 induced cell death via ROS-dependent ER stress and G2/M cell cycle arrest in human prostate cancer cells
Source: BMC Cancer. 2015 Nov 6;15:866. doi: 10.1186/s12885-015-1851-3 (PMC4636884; doi:10.1186/s12885-015-1851-3)
Supplement: Additional file 1: — The anti-cancer effects of WZ35 treatment in PC-3 and DU145 cells. Figures S1, The time-course of cell apoptosis and cell cycle progression in response to WZ35 treatment in PC-3 cells. and S2, The effects of WZ35 treatment in DU145 cells.(DOC 290 kb) [file 12885_2015_1851_MOESM1_ESM.doc]

**Supporting Information**

**Curcumin Analog WZ35 Induced Cell Death via ROS-dependent ER Stress and G2/M Cell Cycle Arrest in Human Prostate Cancer Cells**

Xiuhua Zhang^1,2,3,#^, Minxiao Chen^2,3,#^, Peng Zou^2^, Karvannan Kanchana^2^, Qiaoyou Weng^2,4^, Wenbo Chen^2^, Peng Zhong^2^, Huiping Zhou^2^, Guang Liang^2^,*, Langchong He^1^,**

*^1^ School of Pharmacy, Health Science Center, Xi’an Jiaotong University, Xi’an, Shaanxi 710061, China;*

*^2^* *Chemical Biology Research Center,* *School of Pharmaceutical Sciences,* *Wenzhou Medical Universtiy, Wenzhou zhejiang 325035, China;*

*^3^* *Department of Pharmacy,* *the First Affiliated Hospital of Wenzhou Medical University, Wenzhou, Zhejiang 325035, China;*

*^4^* *Department of Interventional Radiology,* *The* *Fifth Affiliated Hospital of Wenzhou Medical University,* *Lishui, Zhejiang 323000, China*

**Figure S1: The time-course of cell apoptosis and cell cycle progression in response to WZ35 treatment in PC-3 cells.** Cells were treated with WZ35 (10mM) for different time as indicated, and then processed to cell apoptosis analysis using Annexin V-FITC/PI staining (A) or cell cycle analysis using flow cytometer (B).

**Figure S2: The effects of WZ35 treatment in DU145 cells.** (A) The time-course of ROS generation induced by WZ35. DU145 cells were treated with WZ35 (10μM) for different time as indicated, then cells were stained with DCFH-DA and the DCF fluorescence intensity was analyzed with flow cytometry. (B) Representative images for cell apoptosis and cell cycle progression analyzed by flow cytometry. (C) Western blot analysis for CHOP expression. (B-C) Cells were treated with WZ35 (10μM) in the absence or presence of NAC (10mM) for 24h, then cells were processed to cell apoptosis analysis using Annexin V-FITC/PI staining, or cell cycle analysis, or Western blot analysis of CHOP expression as described in methods. (D) The effects of CHOP silence using siRNA approach on cell apoptosis in response to WZ35 treatment. Cells were transfected with Ctrl siRNA or CHOP siRNA for 24h as described in methods. Then the transfected cells were treated with or without WZ35 (10μM) for 24h. Then the cells were processed for western blot analysis for CHOP expression or stained with Annexin V-FITC/PI followed by flow cytometry analysis. The statistic data were presented as mean±S.E from three independent experiments. *,p<0.05, **,p <0.01;.
